# The impact of non-pharmaceutical interventions on the socio-economic and demographic determinants of COVID-19 incidence: A spatial analysis of the pandemic in Toronto, Canada

**DOI:** 10.1371/journal.pone.0347649

**Published:** 2026-05-04

**Authors:** Kevin Siebels, Nicholas H. Ogden, Patricia Turgeon, Juliette Lapeyre, Stéphanie Brazeau

**Affiliations:** 1 Public Health Risk Sciences Division, National Microbiology Laboratory, Public Health Agency of Canada, Saint-Hyacinthe and Guelph, Canada; 2 Groupe de Recherche en Épidémiologie des Zoonoses et Santé Publique (GREZOSP), Faculté de Médecine Vétérinaire, Université de Montréal, Quebec, Canada; 3 Centre de Recherche en Santé Publique (CReSP), Montreal, Quebec, Canada; Fred Hutch Cancer Center: Fred Hutchinson Cancer Center, UNITED STATES OF AMERICA

## Abstract

Socio-economic and demographic variables have been identified as determinants of transmission of, and susceptibility to, COVID-19. In this study, we analyse the heterogeneous impacts of non-pharmaceutical interventions on the socio-economic and demographic (SED) variables driving COVID-19 incidence in Toronto during the 2^nd^, 3^rd^ and 4^th^ waves of the pandemic. Spatial autoregressive models were used to explore associations between COVID-19 incidence and SED variables at neighborhood scale, accounting for vaccination levels. This approach helps clarify how SED factors and vaccine coverage drive COVID-19 incidence and how non-pharmaceutical interventions (NPIs) modulate these factors at neighborhoods’ level, while taking into account the pervious nature of boundaries at these scales to disease transmission due to population mobility, although without directly informing on behaviours, exposure, or vulnerability at individual level. Three distinct models were considered for each of the second, third and fourth COVID-19 waves, from late 2020 to late 2021. Associations highlighted by the models were interpreted with reference to the NPIs implemented. Level of scholarity, income, proportion of the population living alone, average number of children in families, and the proportion of the population whose mother tongue is not an official language showed significant relationships with COVID-19 incidence. Model results were different for each wave, reflecting the unequal impacts of NPIs at different time points, and for different population groups, depending on the nature of interventions and the SED determinants considered. Prioritization of population groups for testing, unequal gathering restrictions, selective closure of economic activities or work-from-home policies led to heterogenous impacts on incidence. The results highlight the unequal burden of the pandemic across populations and likely disparities in occupational exposure driven by SED factors, as well as their evolution with the implementation and lifting of NPIs. Populations with the lowest income and scholarity cumulate the highest risks of exposure and the highest risks of severe disease outcomes. Our results support the development of knowledge based public health surveillance programs integrating both non-communicable and infectious diseases cases, beyond their acute occurrences, along with their socio-economic characteristics.

## Introduction

Following its emergence in late 2019 in Wuhan, capital of Hubei Province, China, COVID-19 rapidly spread globally to become a pandemic. More than 509 million cases and 6.5 million deaths have been reported worldwide up to September 25, 2022 [1].

Non-pharmaceutical interventions (NPIs, i.e., public health measures and policies that do not include vaccinations or therapeutics) are enacted by provincial and territorial governments to manage the pressure on health-care systems and protect population health. The NPIs that are least impactful on society are those that target individuals identified as infected, or those that have likely been exposed to infection — i.e., case detection and isolation, and contact tracing and quarantine. These were not capable of controlling the pandemic alone though [2], and restrictive measures (including closure of schools, universities and non-essential businesses, or restrictions on gatherings etc.) had to be implemented to slow transmission by reducing contact rates, thus preventing additional deaths due to healthcare system capacity being exceeded [3–5].

Vaccines against COVID-19 became available in late 2020, and vaccinations began in Canada in December 2020. However, due to initially limited vaccine supply, vaccines were rolled out to groups considered the most at need due to exposure (such as healthcare workers) or vulnerability (the oldest and those with significant co-morbidities). As vaccine supply increased and vaccines were approved for younger age groups, they were rolled out to the rest of the population with high percentages of the population having received two doses by the end of 2021 [6]. During 2021 and early 2022, a combination of vaccines and NPIs have maintained transmission within the capacity of healthcare until the combination of post-vaccine and post-infection immunity in early 2022 allowed NPIs to be lifted.

Socio-economic and demographic studies of public health issues have provided valuable insights on the factors and circumstances driving the spread of contagious respiratory diseases at populations level. For COVID-19, unequal occupational exposure related to gender [7–10], income [11–13], or education [8,10,14,15] have, for instance, been reported since the early stage of the pandemic. In 2025, Wang et al. [16] analysed how income at dissemination area level —the smallest census geographic unit in Canada—was associated with COVID-19 deaths and diagnoses in Ontario, Canada. Their analysis considered the mediating role of vaccination coverage and the proportion of essential workers, while controlling for several SED confounders. However, because of population mobility, the geographic pattern of infection should also be considered. Incorporating the spatial aspects in SED analysis has indeed improved the understanding of COVID-19 transmission in some studies, but not all [17].

We also argue that evaluating the SED factors impacting the COVID-19 incidence should be performed in light of the NPIs in place. Because specific activities, and consequently population groups, were targeted by NPIs based on their essentialness and exposure risk, histories of public health measures are likely key to understanding why and how the epidemiology varied according to SED groups. In this study, and for the first time to our knowledge, we therefore consider the impact of NPIs on the magnitude of these associations between SED factors and incidence in Toronto neighborhoods, by conducting time period-specific analyses. This retrospective study at neighborhood’s scale intends to assess how statistical relationships between population SEDs and COVID-19 incidence were modulated by NPIs through time, taking into account the spillover of the disease across neighborhoods’ boundaries, although without providing insights on individuals behaviours, vulnerability or exposure.

## Materials and methods

### Data

**Fig 1:**
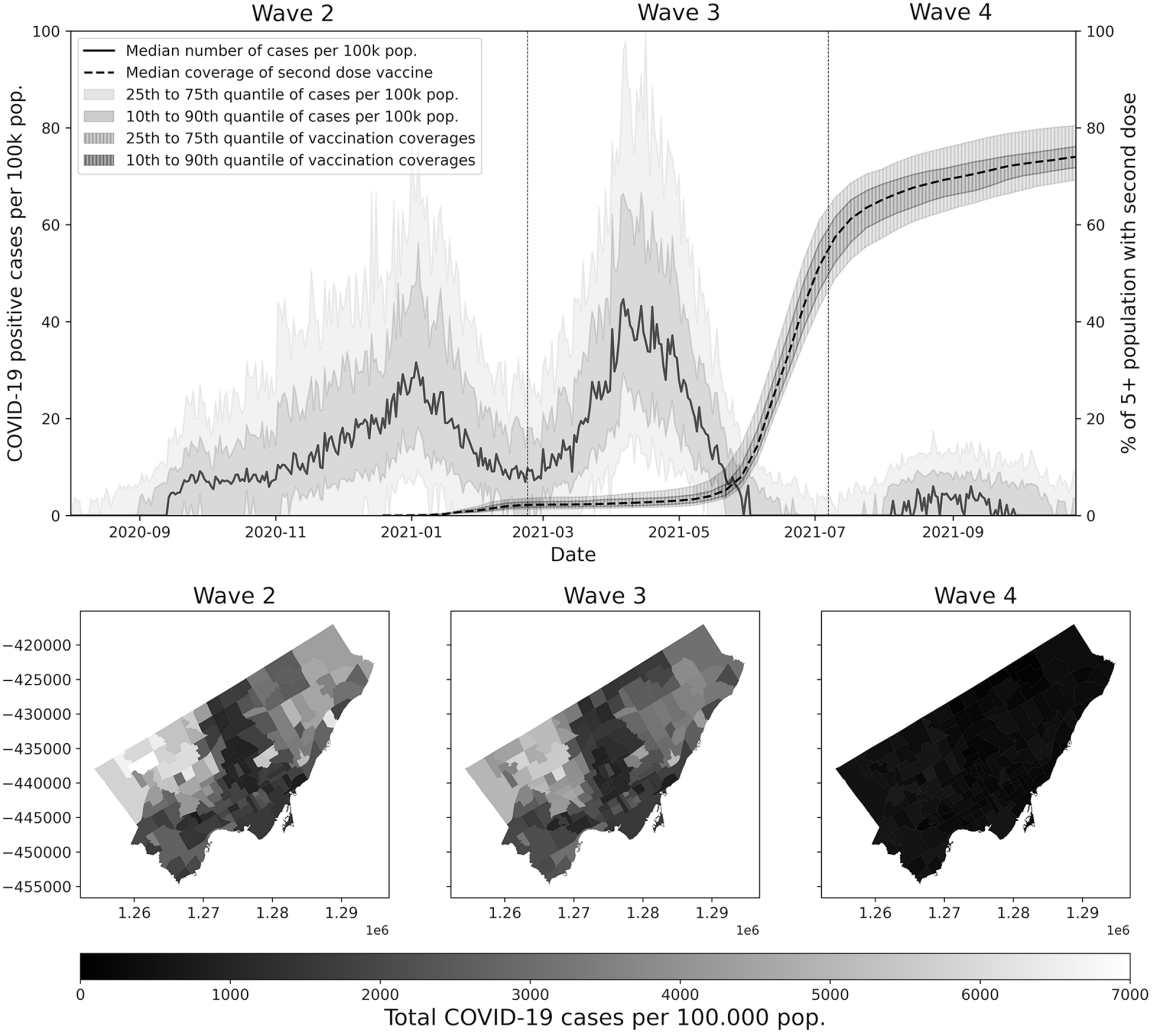
COVID-19 incidence rates in Toronto from second to fourth wave. Based on data from [18]. Top: Rate of positive COVID-19 cases per 100.000 population of Toronto’s neighborhoods and second dose vaccine coverage, from 1 August 2020 to 26 October 2021. Bottom: Spatial distribution of the total incidence of COVID-19 per 100.000 population, during wave 2 to 4. X and Y coordinates are in Lambert Conformal Conic projection (m).

Data on vaccination and COVID-19 surveillance were obtained from the City of Toronto website and downloaded on April 25, 2022, and May 3, 2022, respectively [18]. We did not have access to information that could identify individual participants during or after data collection. Data on the timing of implementation and lifting of different NPIs were obtained from the Government of Ontario [19,20], the City of Toronto [21] and the Canadian Institute for Health Information (CIHI) [22]. Reported confirmed and probable COVID-19 cases were converted to average daily incidence rates per-100k population for the waves 2, 3 and 4 of the pandemic in Toronto from 1^st^ August 2020 to 22 February 2021 (corresponding to wave 2), 23 February 2021 to 7 July 2021 (corresponding to wave 3) and 8 July 2021 to 26 October 2021 (corresponding to wave 4) (Fig 1). Data from the first, fifth and later waves were not used as described above. Data collection methods and case definitions evolved across the time period considered [23–26], including by considering reinfection cases starting in May 2021 [26]. The potential resulting biases on incidence analysis are addressed in the methodology (see Preprocessing).

SED variables were obtained from Statistic Canada [27]. The datasets were aggregated to neighborhood level (using the neighborhood boundaries available from open data of the City of Toronto: City of Toronto, 2022c) to match with the COVID-19 cases and vaccination coverage datasets. Based on previous studies, five themes of SED variables were selected as being those most likely to affect population behavior regarding vaccination and NPIs: age, education, family characteristics, income and language. From these themes, explanatory variables were selected based on the literature cited in the introduction and their potential link with the measures in place during the pandemic. For explanatory variables with correlation *r* > 0.8, the variable considered the most epidemiologically plausible was retained, resulting in 13 explanatory variables (Table 1).

**Table 1:** Statistics of the socio-economic, demographic and vaccination variables.

Explanatory Variables	Mean	Median	Std	Min	Max
Male with Apprenticeship or trades certificate or diploma (%)	0.23	0.22	0.11	0.00	0.64
Male with College, CEGEP or other non-university certificate or diploma (%)	0.88	0.86	0.28	0.19	1.79
Female with Apprenticeship or trades certificate or diploma (%)	0.17	0.15	0.12	0.00	0.64
Female with College, CEGEP or other non-university certificate or diploma (%)	1.21	1.20	0.39	0.13	2.30
With Secondary (high) school diploma (%)	6.18	6.13	1.67	3.43	17.31
With University certificate or diploma below bachelor level (%)	0.48	0.46	0.20	0	1.10
With University certificate, diploma or degree at bachelor level or above (%)	5.32	4.86	2.50	1.53	19.92
65 years and over (%)	15.79	15.39	4.62	0.00	27.97
Average number of children in families (n)	1,70	1,70	0,14	1,35	2,15
Living alone (%)	15.07	13.37	7.38	3.89	40.26
Median amount of income ($)	35583.9	33943.5	9366.6	21292.5	65456.3
Non-official language as mother tongue (%)	41.99	42.62	14.34	11.38	77.43
Vaccination coverage in Wave 2 (2nd dose) (%)	0.76	0.68	0.33	0.20	2.07
Vaccination coverage in Wave 3 (2nd dose) (%)	11.77	11.23	2.51	6.88	19.33
Vaccination coverage in Wave 4 (2nd dose) (%)	71.95	71.89	4.95	57.81	83.05

Explanatory socio-economic, demographic, and vaccination variables extracted from the census of Statistic Canada [27] and Toronto vaccination open data [28]. Variables in percentage (%) express the percentage compared to the total per-neighborhoods population. Education variables refer to the highest obtained certificate, diploma or degree.

### Statistical analysis

A spatial lag model (or ‘spatial autoregressive model’) was used to examine the relationship between SED variables and COVID-19 incidence while accounting for spatial dependence across Toronto neighborhoods. Spatial lag models explicitly incorporate spatial autocorrelation in the outcome variable, i.e., that COVID-19 incidence in a given neighborhood is statistically associated with incidence in nearby neighborhoods through an endogenous structured spatial process.

In the context of this study, COVID-19 transmission extends across neighborhood boundaries due to social interaction and population mobility, creating a spatial interdependence of incidence rates. Unlike non-spatial approaches such as Ordinary Least Squares (OLS), which assume independence across observational units and may yield biased or inefficient estimates in the presence of spatial dependence, the spatial lag model explicitly integrates this interdependence as part of the data-generating process. This allows spatial diffusion mechanisms to be modeled directly rather than treated as residual correlation.

**Fig 2:**
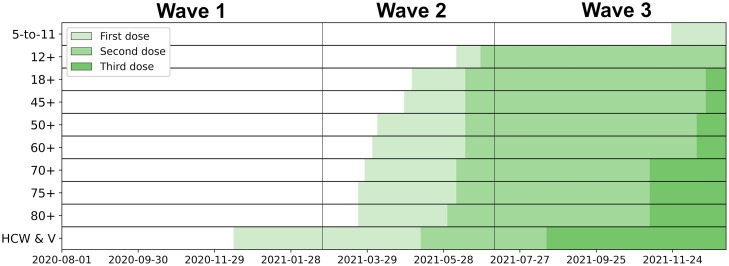
Access to first, second and third doses of COVID-19 vaccines for Toronto’s populations. HCW & V = health-care workers and vulnerable individuals. Based on the CIHI [22] and Ontario regulation laws [19] and announcements [20].

When modeling COVID-19 incidence rates, we ensured that the condition index of the model was below 10 to avoid any substantial multicollinearity [29]. The statistics of the exogenous variables by neighborhood area in Toronto are presented in Table 1 while the evolution of the age-specific vaccination program is shown in Fig 2.

Because the trends of COVID-19 cases are largely driven by population behaviour, the results of the spatial models were analysed along with the NPIs in place. The latter are illustrated in S1 Fig in the Supporting Information for the second, third and fourth waves.

A spatial lag model was used to account for the spatial dependence of COVID-19 incidence rate due to population mobility, social interactions, and transmission processes, along with SED variables and vaccination coverage. The model,


$$\begin{array}{c} y\;=\;\rho \text{Wy }+\text{ X}\beta \;+\;\varepsilon \end{array}$$ (1)

can be rewritten


$$\begin{array}{c} y=(I-\rho W{)}^{-\text{1}}X\beta +(I-\rho W{)}^{-\text{1}}\varepsilon \end{array}$$ (2)

Where *ρ* is the spatial autoregressive coefficient expressing the magnitude of the spatial interdependence of a spatial-unit outcome with neighbouring outcomes. *W* is the spatial weights matrix, expressing the importance of neighbors in the modeled process. The parameter *y* is the *n* x 1 vector of the dependent variable with *n* observations. *X* is the matrix of explanatory variables, *β* is the vector of regression coefficients, *ε* is the error term, and *I* is the identity matrix. The model is equivalent to the ordinary least squares (OLS) when *ρ* is zero.

From Equation (2) the spatial multiplier *(I –* ρ*W)*^*-1*^ can be developed as:


$$\begin{array}{c} (I-\rho W{)}^{-\text{1}}=I+\rho W+\rho^{\text{2}}W^{\text{2}}+\rho^{\text{3}}W^{\text{3}}+\ldots \end{array}$$ (3)

, reflecting successive diffusions to neighbors, neighbors of neighbors, etc.

From Equation (3) can be calculated the direct, indirect and total impacts of a considered covariate on incidence rates. The direct effect measures the average change of the dependent variable within a spatial unit resulting from a change in a covariate in that same unit, accounting for spatial feedback loop from neighboring units. The indirect effect, or ‘spillover’, measures the average change of the dependent variable in neighboring units, resulting from a change in a covariate in a given unit, as this change propagates through the spatial dependence structure. The total effect is the cumulated effect of the direct and indirect effects and represents the overall system-wide response to a change in a covariate.

The distance-weight matrix W was computed using the Minkowski distance formula that generalize distance metrics, including the Euclidean and Manhattan distances:


$$\begin{array}{c} D_{\alpha ,p}(x,y)=\;{\left(\sum_{i=\text{1}}^{n}{\left|x_{i}-y_{i}\right|}^{p}\right)}^{\frac{\text{1}}{p}}\; \end{array}$$ (4)

Where *D* is the computed distance, and *i* indexes the dimensions of the coordinates vector of the pair of points *x* an *y,* which represent two-dimensional coordinates in this context. The parameter *p* ∈ *[1,∞)* defines the norm used in the distance calculation. The Minkowski distance formula encompasses both the Euclidean distance p = 2; representing free movement in all directions) and Manhattan distance (p = 1; to express movements constrained by a grid-like road network). Finally, a decaying weight function was applied to the computed distance, using an inverse exponent parameter *α*:


$$\begin{array}{c} w(x,\;y)=d^{-\alpha } \end{array}\;$$ (5)

Where *α* = −1 results in a linear decay with distance, and lower values (e.g., α = −2) produce a steeper decay, reducing the weight of more distant spatial units compared to nearby ones. The resulting weights were then standardized by dividing each by the sum of all weights so that they sum to one.

Separate spatial lag models were developed for the second, third, and fourth waves using the 13 explanatory SED variables and vaccination coverage (Table 1) to model the average number of positive cases per 100,000 population per wave. In this study, instead of applying a strict distance limit to define spatial neighbors, neighborhoods’ relative influence were defined by the α parameter in the decaying weight function (3), ranging from 0.5 to 3 with a step of 0.1. The best model was selected based on their Log Likelihood. The Likelihood-Ratio χ^2^ test (LR χ^2^) of the spatial lag models against the nested OLS model [30,31] was used to assess the relevance of using spatial against non-spatial approaches.

### Preprocessing

Because the modeling approach uses maximum likelihood under an assumption of normality of residuals to assess the statistical relationships with the considered covariates, we ensured residuals followed a normal distribution by applying a per-wave log transformation of the incidence data.

Additionally, because the three pandemic waves considered differ substantially in magnitude (Fig 1), the resulting wave-specific model coefficients would directly reflect these contrasts, making comparisons of the relative impacts of the covariates across waves more difficult. To make the coefficients more comparable across differences in incidence magnitude and to better highlight the impact of NPIs, the incidence rates were therefore standardized by subtracting their mean and dividing by their standard deviation. These preprocesses also allow to address the possible biases that may arise from the evolving definition of COVID-19 positive cases that potentially affected the waves magnitudes.

Finally, although the same SED variables were used across the three waves considered, these variables are expressed in different units (e.g., “%” or “$”) and may exhibit markedly different distributions and variances across Toronto neighborhoods; A 1% change may represent a realistic difference between neighborhoods for the proportion of the population aged 65 years and over (Table 1), but would be unrealistic for the proportion of ‘Males with an apprenticeship or trades certificate or diploma’, given its distribution (Table 1). Conversely, while a one-unit change represents a substantial difference for the latter, a one unit change in median household income, expressed in dollars ($), would be negligible. Standardizing the SED variables places their magnitude and variance on a common scale, facilitating comparisons of their relative importance across waves, although at the cost of making their effects on incidence less directly interpretable.

Unlike the SED variables, whose distributions remain unchanged across the three waves considered, vaccination coverage increased substantially over time, leading to marked differences in its magnitude and variance across waves (Table 1). Therefore, vaccination coverage was standardized separately for each wave, with standard deviations being 0.4, 2.6, and 4.6 percentage points for waves 2, 3, and 4, respectively (Table 1). Although wave-specific standardization accounts for the evolving scale of vaccination coverage over time and allows its relative importance to be assessed consistently alongside other covariates within each wave, direct comparisons of its coefficient magnitudes across waves must be interpreted cautiously.

## Results

The performance of the best Spatial Lag models and their comparison with the corresponding nested OLS models are summarized in Table 2.

**Table 2:** Statistics of the best spatial lag models and their respective nested OLS model from wave 2 to wave 4.

	Wave 2	Wave 3	Wave 4
Log Likelihood (OLS)	−90.306	−91.418	−160.943
Log Likelihood (Lag)	−81.315	−86.497	−155.811
Log Likelihood χ^2^ (Lag vs OLS)	17.98 (p < 0.001)	9.841 (p = 0.002)	10.263 (p = 0.001)
BIC (OLS)	249.492	251.715	390.766
BIC (Lag)	236.429	246.794	385.422
Moran I (Lag)	−0.005	0.007	−0.004
Direct Effect	1.013	1.020	1.021
Indirect Effect	2.486	3.403	3.706
Total Effect	3.499	4.423	4.728
Autoregressive coefficient (rho)	0.714 (P < 0.001)	0.774 (P < 0.001)	0.788 (P < 0.001)

Log Likelihood: Log Likelihood of the spatial lag and OLS models. Log Likelihood χ^2^: Log Likelihood χ^2^ (ratio) test of the lag models compared to their respective nested OLS model. BIC: Bayesian Information Criterion. Moran I: Moran autocorrelation Index of models’ residuals. Direct Effect: Direct relative effect of the covariates locally, accounting for the feedback loop. Indirect Effect: Estimated average spillover effect on neighboring spatial units

The Log Likelihoods of the spatial lag models from wave 2 to wave 4 consistently show a better fit compared to the OLS nested models, supporting the hypothesis of a spatial structure in the disease transmission. This is confirmed by the likelihood χ^2^ tests that strongly rejects the null hypothesis of spatial independence for the three waves. In the meantime, the Bayesian Information Criteria (BIC) confirm the higher benefit in terms of predictions over the increasing complexity caused by the addition of the spatial autoregressive coefficient in the model, further supporting the integration of the spatial dependence when modeling COVID-19 incidence rates. The small values of the Moran I on residuals additionally underscores that the latter has been effectively captured for all three waves.

Accounting for endogenous spatial feedback increases the average local impact, i.e., the direct effect of covariates, by approximately 1.3% to 2.1% relatively to a non-spatial specification. In contrast, the average spillover effect on other neighborhoods, i.e., the indirect effect, amounts to approximately 3.5 to 4.7 times the purely local non-spatial effect, indicating that most of the covariate impact operates through spatial propagation rather than through local feedback alone. This underlies how spatial dependence operates primarily through inter-neighborhood diffusion rather than self-reinforcement, which is consistent with the high autoregressive coefficients that express the strength of endogenous spatial dependence in the outcome variable, with values increasing monotonically from 0,714 for the second wave to 0.788 for the fourth one.

The drop of the model’s ability to predict COVID-19 incidence rates during the fourth wave (Table 2) coincides with the lifting of most NPIs during this time period (S1 Fig), suggesting an association between the explanatory power of the selected SED and the socio-economic compartmentalization of Toronto’s population. A complementary analysis was performed to assess the statistical relationship between the ability of our approach to model COVID-19 incidence and the stringency of the NPIs enacted. The method and results are described in the Supporting information, S1 Appendix. The significantly positive Spearman correlation between the performance of the model, expressed as Log Likelihood, and most (but-not-all) per-sector stringencies, and with the global mean NPI stringency, support the interpretation of an association.

The coefficients of the models for the explanatory variables considered for waves 2, 3 and 4 and for the spatially lagged component are presented in Table 3.

**Table 3:** Results of the spatial lag models for the three waves considered.

	Wave 2			Wave 3			Wave 4		
	Coeff	p	SE	Coeff	p	SE	Coeff	p	SE
Female with Apprenticeship or trades certificate or diploma	0.009	0.856	0.050	0.065	0.207	0.052	0.153	0.072	0.085
Female with College, CEGEP or other non-University certificate or diploma	−0.011	0.872	0.068	−0.002	0.983	0.070	0.062	0.596	0.118
Male with Apprenticeship or Trades certificate or diploma	**0.196**	**0.000**	**0.044**	**0.188**	**0.000**	**0.045**	0.146	0.052	0.075
Male with College, CEGEP or other non-University certificate or diploma	**0.096**	**0.037**	**0.046**	0.082	0.086	0.048	**0.187**	**0.021**	**0.081**
With Secondary (high) school diploma	−0.018	0.782	0.066	0.077	0.254	0.068	0.102	0.375	0.115
With University certificate or diploma below bachelor level	−0.053	0.298	0.051	0.045	0.398	0.053	−0.105	0.230	0.087
With University certificate, diploma or degree at bachelor level or above	**−0.221**	**0.023**	**0.097**	**−0.298**	**0.005**	**0.106**	−0.093	0.561	0.160
65 years and over	**−0.161**	**0.003**	**0.054**	−0.106	0.091	0.063	−0.082	0.310	0.081
Average number of children in families	**0.202**	**0.000**	**0.056**	0.067	0.275	0.061	0.019	0.849	0.099
Living alone	**0.257**	**0.001**	**0.078**	**0.236**	**0.003**	**0.081**	0.174	0.194	0.134
Median amount of income	**−0.462**	**0.000**	**0.086**	**−0.438**	**0.000**	**0.091**	0.128	0.395	0.151
Non-official language as mother tongue	**0.189**	**0.014**	**0.077**	0.011	0.889	0.080	−0.081	0.522	0.126
Vaccination coverage (2^nd^ dose)	**0.152**	**0.004**	**0.053**	−0.100	0.202	0.078	**−0.335**	**0.000**	**0.088**
Autoregressive Coefficient (ρ)	**0.714**	**0.000**	**0.201**	**0.774**	**0.000**	**0.159**	**0.788**	**0.000**	**0.149**

Coefficients (Coeff), p values (p) and Standard Errors (SE) of the variables considered in the spatial lag models. Coefficients express the magnitude of the statistical relationships between the standardized explanatory variables and the log-transformed standardized incidence rate, at neighborhood level. Significant variables are highlighted in bold.

From the results presented in Table 3, we identified 10 factors showing significant relationships with the incidence rates in Toronto neighborhoods. The number of significant factors decreased across the three waves considered, which coincides with the declining model fit reported in Table 2. These coefficients reflect the immediate local impact of the standardized explanatory variables on the log-transformed standardized incidence rates wave-wise. They are used in the rest of this paper as they allow to compare the intrinsic association strength of SED variables with incidence rates, without the amplification caused by the transmision network expressed by the average direct and indirect effects reported in Table 2. Accordingly, the direct, indirect, and total effects of the standardized covariates on incidence rates, expressed in % difference, are provided in S2 Appendix in the Supporting Information to facilitate a more comprehensive interpretation. Throughout the remainder of this paper, coefficient magnitudes are classified as ‘low’, ‘moderate’, ‘high’, or ‘very high’, corresponding to values between 0 and 0.25, 0.25 and 0.5, 0.5 and 0.75, and 0.75 and 1.0, respectively.

The proportion of the population aged 65 and over were significantly and negatively associated with lower incidence during the second wave (β_k_ = −0161; p = 0.003). The sign of the relationship was consistent across the three waves considered and its magnitude stayed low and decreased, along with its significance, from wave 2 to wave 4.

Scholarity-related variables show significant associations, although the coefficients and levels of significance differ by sex and education level. The proportion of males holding apprenticeship or trades diplomas shows a low positive and significant association with incidence during second (β_k_ = 0.196; p = 0.000) and third waves (β_k_ = 0.188; p = 0.000) and was slightly beyond significance threshold only during the third wave (β_k_ = 0.146; p = 0.052). In contrast, the proportion of females with these education levels was not significantly associated.

A similar pattern is observed for the population holding a college, CEGEP, or other non-university certificate or diploma. The proportion of males in this group shows a low but significant positive association with incidence in waves 2 (β_k_ = 0.096; p = 0.037) and 4 (β_k_ = 0.187; p = 0.021), and a marginally significant association in wave 3 (β_k_ = 0.082; p = 0.086). Again, no significant relationships were identified for females in this education category across any of the three waves.

Meanwhile, the share of the population holding a university certificate, diploma, or degree at the bachelor level or above shows significant negative low and moderate associations with incidence during waves 2 (β_k_ = −0.221; p = 0.023) and 3 (β_k_ = −0.298; p = 0.005), respectively. Taken together, these findings support the hypothesis of differential gender- and education-related occupational exposure.

Regarding household composition, the proportion of the population living alone was positively and significantly associated with incidence rate during wave 2 and 3, its magnitude decreasing slightly from β_k_ = 0.257 (p = 0.001) to β_k_ = 0.236 (p = 0.003). On the other hand, the average number of children in families also showed a low positive and significant association during the second wave (β_k_ = 0.202; p = 0.000), its magnitude and significance decreasing afterward. “Children” here refers to persons living with their parent(s), without consideration of their age.

There was a moderate negative significant relationship between income and COVID-19 incidence during the second (β_k_ = −0.462; p = 0.000) and third (β_k_ = −0.438; p = 0.000) waves. Likely related to income and occupational exposure too, the proportion of the population with a non-official language as their first language had a low positive association with incidence during the second wave (β_k_ = 0.189; p = 0.014).

Besides NPIs and SEDs, vaccination coverage shows a low but positive association with incidence in wave 2 (β_k_ = 0.152; p = 0.004), and a moderate negative association in wave 4 (β_k_ = −0.335; p = 0.000). It is important to note that the coefficient magnitudes across the three waves correspond to different “one-unit” changes in vaccination coverage, because the explanatory variables were standardized separately for each wave (Table 1) while vaccination rollout progressed over time (Fig 1). To make the coefficients from wave 2 and wave 4 comparable, the coefficient for vaccination coverage in wave 4 must be divided by 14.8 so that the underlying “one-unit” change corresponds to the same absolute change in vaccination coverage.

Although the autoregressive coefficient is an endogenous structural parameter, on the contrary of SED and vaccination covariates that are exogenous, it plays an important role in the magnitude of the incidence rates observed by expressing the dependence of the local incidence rate of a given neighborhood to the incidence rates of its neighbors. It has the highest and most significant positive associations for the three waves considered, increasing monotonically from a high magnitude during the second wave (β_k_ = 0.714; p = 0.000), to reach a very high magnitude (β_k_ = 0.788; p = 0.000) for the fourth one, underscoring again the importance of considering spatial patterns of transmission in models.

## Discussion

### Older age groups

The negative relationships between incidence and the proportion of people in older age groups may be driven by the recognition of the risks of developing severe symptoms. Consequently, those 65 years and over may had more diligent compliance to public health measures and higher avoidance of exposure [32]. During the third wave, more stringent closures of businesses and recreational activities (S1 Fig and S1 Appendix) likely reduced contacts with younger age groups, diminishing the relative influence of older populations on incidence.

Older age groups were also the first to have access to vaccination. The administration of first and second doses for those aged 60 years old and over started on 2 April 2021 and 4 June 2021, respectively (Fig 2). Consequently, populations 65 + were the age groups with the highest levels of immunization when the Delta variant-dominated fourth wave began. On 1^st^ July 2021, two-dose vaccination became also mandatory for staff in long-term care homes. This interpretation aligns with the higher positive correlation observed during the third wave between the share of population being 65 and over and the mean vaccination coverage at this time of the pandemic (S2 Fig). As of 30 May 2021, about 66% of the staffs were recorded as fully immunized while the coverage was of about 97% for the residents [33].

### Scholarity

The proportion of males with apprenticeship or trades certificates or diplomas had a significant positive relationship with incidence in waves 2 and 3. A possible cause is that essential construction projects continued throughout the pandemic while other sectors were more severely limited (S1 Fig). In Canada, the work-from-home capacity of this sector is also among the lowest of all sectors, with less than 15% of workers being able to do so [14]. Similarly, Lan et al. 2020 found that construction laborers have higher risks of transmission in a work-related context [34]. This is also consistent with the per-sector incidence comparison in Allan-Blitz et al. 2020, with the construction sector reported as having the highest and second highest incidence among asymptomatic and symptomatic people tested in the US, respectively [35]. The absence of any relationship with the female equivalent also suggests a contrast of occupational exposure due to gender. This hypothesis is supported by the 7:1 ratio of males to females employed in the construction industry in Ontario [36].

Although an association with NPIs is not put forward, differences in occupational exposure is also proposed to explain the contrasted statistical associations estimated between male and female populations with college, or other non-university certificate or diploma. This aligns with other studies in Canada and Europe reporting similar risks inequalities between men and women, and between education levels [7–10]. The significant low and moderate significant positive associations estimated between the incidence rate of COVID-19 and the proportion of the population with university certificate, diploma or degree at bachelor level or above, aligns with these studies too.

### Household composition

Starting on 23 November 2020, an exemption was introduced for lockdowns and gathering restrictions for people living alone to be able to gather with other households and circumvent the mental burden of complete social isolation. While only significant during the first two waves considered, this likely explains the positive relationship of this population group with incidence.

In contrast, the average number of children in families also showed a low but significant positive relationship with incidence during the second wave. We interpret these results as related to the changes in the criteria for accessing COVID-19 tests. The testing eligibility were progressively extended to asymptomatic individuals starting at mid-first wave on 29^th^ May 2020 [37], but with a particular focus on individuals in schools and child care immediately before the third wave, on 1^st^ February 2021 [38]. Consequently, asymptomatic children and teenagers, while mainly “unseen” during the first and second waves, were more likely detected during the third and fourth ones, leading to self-isolation, contact-tracing, and better contact-case management. Additionally, first vaccine doses were first administered in Toronto to 12 + population while the third wave was mostly over, on 23 May 2021 (Fig 2) [39], further reducing the magnitude and significance of the association during wave 4. It is noteworthy to mention that vaccination coverage remained lower for younger age groups during fourth wave and later [40]. In the meantime, schools were comparatively more frequently open for in-person learning during the second wave. We therefore interpret the impacts of this factor as a combination of higher incidence due to exposure in schools, limited case detection due to restrained eligibility to testing, and late vaccination of children.

### Income

Along with education and gender, income has often been reported as a critical factor to understand SED impacts on COVID-19 incidence [11–13], which is supported by our study with significant negative moderate relationships observed during second and third waves. Messacar et al. [13] highlighted the systematic increase of the ability to work from home with higher earning, ranging from below 10% for the lowest decile of income to more than 50% for the highest one. On the other hand, a sector-blind income comparison showed that women are systematically more likely to occupy work-from-home-compatible jobs compared to men of similar income [13].

### Immigration and visible minorities

Neighborhoods with higher proportions of people having a non-official language as their mother tongue had significantly higher incidence during the second wave. This factor is interpreted here as a proxy for multiple determinants influencing the occupational exposure, including immigration status and belonging to visible minorities. This result is indeed consistent with the higher COVID-19 incidence reported in ethno-racial groups, with the exception of East Asian, as compared to the total population in Ontario [41–43]. These observations also match with other inequalities reported in the literature and with the variables already discussed above; immigrants who arrived in Canada as adults are overrepresented in the health care and nursing occupations [44], which is known for being a sector with very high exposure to COVID-19. In addition, economic data show a substantial income gap between white workers and visible minorities or immigrants (Canadian-born or first-generation immigrants; [45]).

### Vaccination

Vaccination presents a seemingly surprising low but significant positive relationship with incidence during the second wave. During the last third of the second wave, vaccines were only administrated to highly-exposed health-care workers and the most vulnerable individuals (Fig 2). Consequently, we suggest that vaccination coverage should, at this point of the pandemic, be interpreted as a marker of particularly exposed communities, rather than better immunized ones. This vaccination strategy evolved then to include an ever-increasing part of the population. The vaccination coverage during the fourth wave shows a significant negative relationship, supporting the effectiveness of vaccination for the more than 70% of the 5 years and older by the middle of wave 4 (Fig 1, Top).

### Impact of neighboring incidence and the direct and indirect spatial effects

The incidence in surrounding neighborhoods exhibits the highest and most statistically significant association with local incidence rate across the three waves considered. This strong spatial dependence of COVID-19 incidences, expressed here by the autocorrelation coefficient ‘*ρ*’ in Equations 1, 2 and 3, aligns with other studies that highlight the improved performance of models that account for spatial transmission and underscores the potential bias that may occur in spatially-blind analyses. In this study, its rise across the time period considered reveals how the transmission system becomes more spatially interdependent, increasingly driven by nearby incidences. In parallel, the models show major indirect effects during the three waves considered (Table 2), revealing how much and how far local changes propagate across the spatial transmission network, i.e., spilling over to other neighborhoods.

Having both the autocorrelation coefficient and the indirect effect increasing monotonically across the period considered pinpoint how the spatial component of disease transmission is intensifying both in strength and in reach, leading to a more network-driven regime. This is consistent with increasing inter-area connectivity and reduced spatial containment of the disease.

### Impacts of NPIs on the socio-economic pattern of incidence

The models show a decline in performance along with the number of NPIs in place during the three waves considered (Table 2). A plausible cause is that, as NPIs loosened and economic activity increased, the number of determinants influencing COVID-19 incidence decreased. This likely reduced the capacity of the 13 selected variables to capture SED dependencies. Conversely, partial or total business closures and lockdowns during second and third waves may have reduced social connections between population groups, increasing the social compartmentalization of transmission. This interpretation is supported by significant positive associations between the stringency of the NPIs and the ability of the approach to model COVID-19 incidence (S1 Appendix) as well as by the growing interdependence of neighborhoods incidences, pinpointed by the increasing magnitude of the spatial autocorrelation and indirect effects.

## Limitations

There are limitations to the study. Lorant et al. [46] highlighted how biases affect socio-economic studies because individuals from lower socio-economic groups are less likely to participate in health surveys. Similarly, because surveillance for COVID-19 cases depended in part on volunteering for testing, the results presented may be biased due to variations in the likelihood individuals or groups underwent COVID-19 tests. There is also a risk of ecological fallacies in the analysis here, with observations at group-level potentially leading to wrong conclusions at individual level. Because of the retrospective and ecological nature of this approach, our results cannot be interpreted in terms of individual-level mechanisms and cannot support direct causality without careful assumptions. The models also consider neighborhoods interdependence spatially and temporally homogenous within each of the three time-periods considered.

However, we argue that many references are now available to inform on unequal exposures, and systematic collection of data on the NPIs used would tend to support interpretations of the model results. Further research on the outcomes of the NPIs would, however, be of great value to informing future decisions on their use.

While the analysis presented in this study interprets the COVID-19 incidence through the influence of NPIs on population exposure, other cofactors reported in the literature may increase susceptibility to COVID-19 (see introduction). Particularly, chronic health conditions increasing the likelihood of COVID-19 infection and severe outcomes. In the context of the pandemic, the unequal burden of health is thus at least twofold for population groups of lower income, with higher rates of chronic disease and higher susceptibility to- and severity of COVID-19 infection. Because socio-economic determinants play a key role in driving both COVID-19 susceptibility (due to initial chronic health conditions) and exposure (due to the economic activity and NPIs), the impact of the heterogeneous exposure considered in this study is likely exacerbated.

This study is also subject to the modifiable areal unit problem (MAUP), as spatial statistical results depend on the scale and zoning of the areal units employed. Aggregating COVID-19 incidence and SED variables at the neighborhood level may mask within-area heterogeneity or alter the magnitude of spatial dependence, thereby influencing estimated direct and indirect effects. Neighborhoods were used because it was the smallest spatial unit available for incidence and vaccination data, hence offering the best spatial resolution available, and results should be interpreted as scale-specific.

## Conclusion

NPIs were enacted by governments to minimize SARS-CoV-2 transmission and protect health care systems and the health of the populations. Because of their heavy economic and social burden and because several services are essential and had to remain active, populations were unequally affected by these measures. This study presents how the rate of COVID-19 cases in Toronto’s neighborhoods are related to their SED variables and how these relationships changed across the three waves considered because of the evolving NPIs. Key findings of this study are evidence for associations between age, scholarity, household size, income and ethnicity of Toronto populations, and COVID-19 incidence. Prioritizing population groups for testing, unequal gathering restrictions, selective closure of economic activities or work-from-home policies also impacted incidence. Together these findings underline the importance of SED on vulnerability to infectious diseases such as COVID-19, and also the heterogenous benefits of NPIs on different population groups.

The spatial lag model led to significant improvements in modeling the rate of COVID-19 cases, compared to the OLS model. The spatial model obtained good fits for the second and third waves, but fit less well for the fourth wave. This is likely due to a resumed economic activity with the partial lift of the NPIs in place, and higher vaccine coverage. Indeed, the unequal capacity of the various work areas to undertake social distancing and the consequent social compartmentalization of SED groups, combined with initially highly selective access to vaccines, likely exacerbated the contrasting exposure to COVID-19 among the population groups during the earliest stages of the pandemic. The subsequent roll out of vaccination to the wider population then generalized the decreasing incidence of COVID-19 cases, leading to a progressive drop of significance of the SED determinants.

The synergistic relationship between socio-economic determinants, COVID-19 and non-communicable diseases exacerbates the heterogeneous burden of health conditions among socio-economic population groups. Fortunately, increasing evidence identifying the socio-economic and health driving factors allows for a better understanding of the population vulnerabilities. As underscored in [47], this supports holistic knowledge-based public health surveillance programs that integrate chronic and infectious diseases beyond their acute occurrences, along with their socio-economic characteristics.

## Supporting information

S1 Fig Timelines of the NPIs implemented during second, third and fourth waves in Toronto for various economic sectors. Increased hatch density corresponds to more severe restrictions, diminishing the attendances or gatherings allowed. The figure is based on information on NPIs gathered by the Canadian Institute for Health Information [22], Ontario regulation laws [19] and Government announcements [20].
(TIF)

S1 Appendix Statistical relationship between the stringency of the enacted NPIs and the models. (PDF)

S2 Appendix Direct, indirect and total effects on incidence rates. (PDF)

S2 Fig Pearson correlation matrix of the explanatory variables used in the spatial lag models. (TIF)

S1 Dataset Daily COVID-19 incidence per neighborhood. (CSV)

## Abbreviations

ADE: Average Direct Effect
AIE: Average Indirect Effect
ATE: Average Total Effect
BIC: Bayesian Information Criterion
CIHI: Canadian Institute for Health Information
LR Chi ^2^: Likelihood-Ratio Chi-Squared
MAUP: Modifiable Areal Unit Problem
NPI: Non-pharmaceutical Interventions
OLS: Ordinary Least Square
SED: Socioeconomic and Demographic.
