# Supplementary figures and images for: The impact of non-pharmaceutical interventions on the socio-economic and demographic determinants of COVID-19 incidence: A spatial analysis of the pandemic in Toronto, Canada
Source: PLoS One. 2026 May 4;21(5):e0347649. doi: 10.1371/journal.pone.0347649 (PMC13138614; doi:10.1371/journal.pone.0347649)

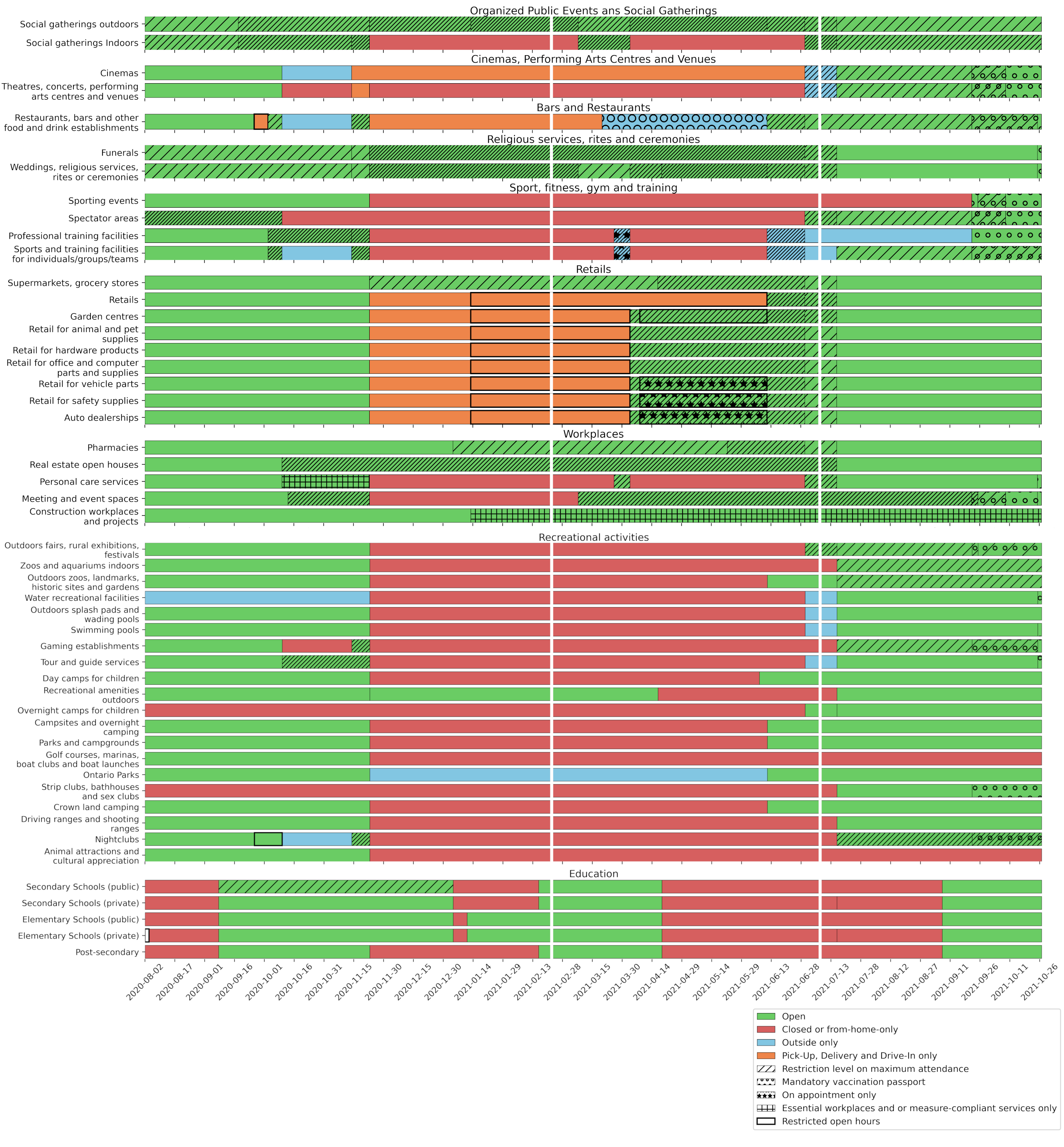

Supplement: S1 Fig — Increased hatch density corresponds to more severe restrictions, diminishing the attendances or gatherings allowed. The figure is based on information on NPIs gathered by the Canadian Institute for Health Information [22], Ontario regulation laws [19] and Government announcements [20]. (TIF) [file pone.0347649.s001.tif]

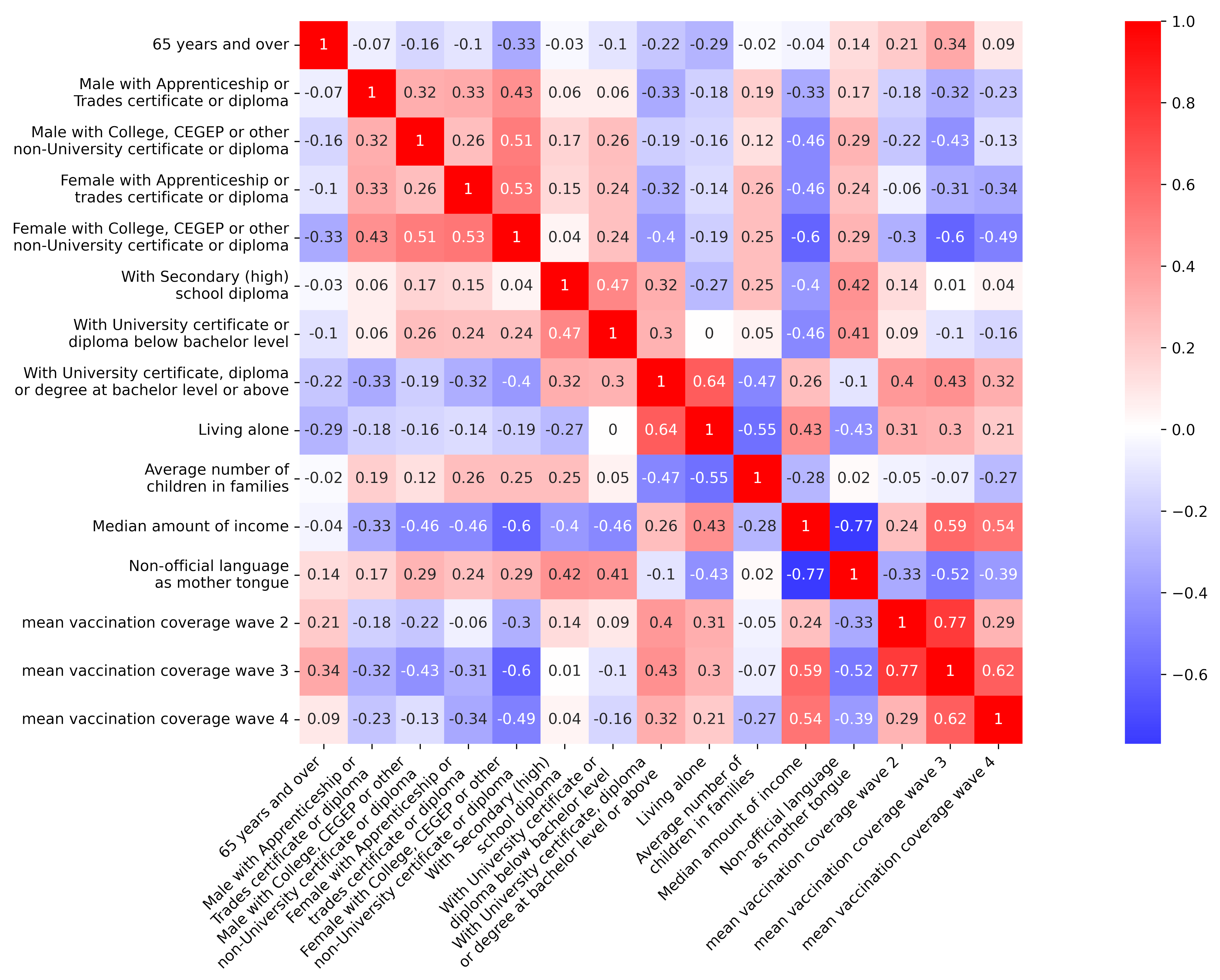

Supplement: S2 Fig — (TIF) [file pone.0347649.s004.tif]
