# Supplementary material for: The impact of non-pharmaceutical interventions on the socio-economic and demographic determinants of COVID-19 incidence: A spatial analysis of the pandemic in Toronto, Canada
Source: PLoS One. 2026 May 4;21(5):e0347649. doi: 10.1371/journal.pone.0347649 (PMC13138614; doi:10.1371/journal.pone.0347649)
Supplement: S1 Appendix — (PDF) [file pone.0347649.s002.pdf]

S1 Appendix. Statistical relationship between the stringency of the enacted NPIs and the models

A four-steps approach was designed to assess the statistical association between the stringency of the enacted NPIs and the ability of the approach to model the COVID-19 incidence:

1. The stringency of the NPIs enacted on each of the economic sectors, illustrated in Fig A, was estimated using an ordinal (semi-quantitative) scale ranging from 0 (no NPI enacted) to 1 (strongest stringency observed). The average of all per-sector stringency level was calculated and used as an estimate for the global stringency of the NPIs enacted.

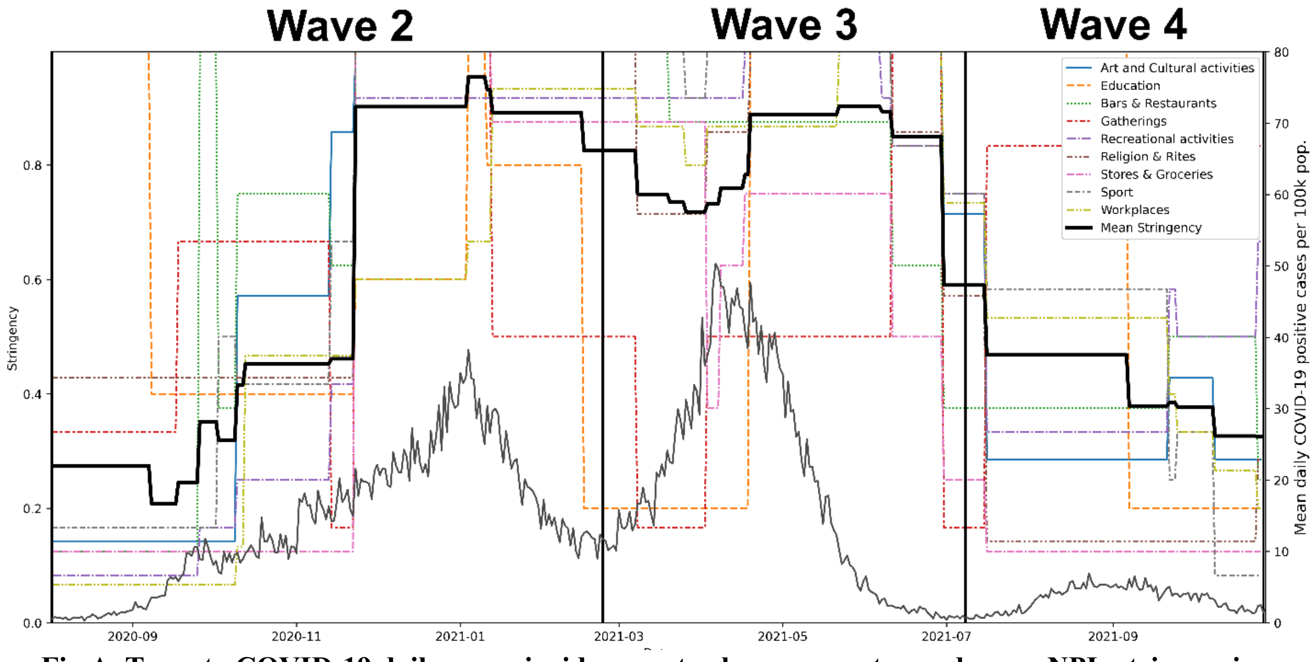

Fig A. Toronto COVID-19 daily mean incidence rate along per-sector and mean NPIs stringencies.

2. The average COVID-19 incidence rate per neighborhood was then computed with a 28 days time-step (instead of per-wave basis, as in the manuscript).
3. A best-fit spatial lag model was then computed for each 28-days step using the same approach as in the main analysis of this study (see 2.2 Statistical analysis in the article)
4. The statistical association between the sector-specific and global stringencies (step 1 and 2), and the performance of the spatial lag models in modeling the COVID-19 incidence (step 3), expressed as adjusted-R^2, was then computed using Spearman correlation (ρ). The results are summarized in Table A.

Table A. Spearman correlation between models performance and NPI's stringency.

	Art and Cultural activities	Education	Bars & Restaurants	Gatherings	Recreational activities	Religion & Rites	Stores & Groceries	Sport	Workplaces	Mean Stringency
Models Adj. R^2	0.53*	-0.029	0.65**	-0.21	0.48*	0.68**	0.59*	0.56*	0.33	0.51*

Spearman correlations between models performance, expressed as Log Likelihood, and NPIs' stringency, both computed at 28-days interval. * : p<0.05; ** : p< 0.01.
