# Supplementary material for: The impact of non-pharmaceutical interventions on the socio-economic and demographic determinants of COVID-19 incidence: A spatial analysis of the pandemic in Toronto, Canada
Source: PLoS One. 2026 May 4;21(5):e0347649. doi: 10.1371/journal.pone.0347649 (PMC13138614; doi:10.1371/journal.pone.0347649)
Supplement: S2 Appendix — (PDF) [file pone.0347649.s003.pdf]

S2 Appendix: Direct, indirect and total effects on incidence rates

The formula to calculate the relative change of incidence rates caused by a one-unit change of a given explanatory variable, accounting for the spillover effects, can be written as follow:

$$\%\Delta y_{direct} = \left[\exp\left(\sigma_{\log y} \cdot ADE_k\right) - 1\right] \times 100$$

$$\%\Delta y_{indirect} = \left[\exp\left(\sigma_{\log y} \cdot AIE_k\right) - 1\right] \times 100$$

$$\%\Delta y_{total} = \left[\exp\left(\sigma_{\log y} \cdot ATE_k\right) - 1\right] \times 100$$

Where *%Δy* is the direct, indirect and total change, expressed in %; $\sigma_{\log y}$ is the wave-specific standard deviation of the incidence rate after log transformation; and ADE_k, AIE_k and ATE_k are the average direct, indirect and total relative effect of a one-unit-change of the variable β_k. In Table B, equations above have been applied to calculate the relative change of incidence rate per standard deviation of the explanatory variable. The reader can refers to the standard deviation values in Table 1 in the main text to calculate changes as per the original variable unit (i.e. *%*, *$* or *n*).

Table B. % change of incidence rate caused by SED variables.

	Wave 2			Wave 3			Wave 4		
	**ADE**	**AIE**	**ATE**	**ADE**	**AIE**	**ATE**	**ADE**	**AIE**	**ATE**
Female with Apprenticeship or trades certificate or diploma	0.52	1.27	1.79	3.29	11.39	15.05	6.62	26.18	34.53
Female with College, CEGEP or other non-University certificate or diploma	-0.62	-1.51	-2.11	-0.07	-0.25	-0.32	2.66	9.99	12.92
Male with Apprenticeship or Trades certificate or diploma	**11.77**	**31.38**	**46.84**	**9.81**	**36.66**	**50.06**	6.30	24.82	32.69
Male with College, CEGEP or other non-University certificate or diploma	**5.58**	**14.26**	**20.64**	4.17	14.60	19.37	**8.16**	**32.92**	**43.76**
With Secondary (high) school diploma	-1.02	-2.49	-3.49	3.92	13.618	18.14	4.40	16.90	22.05
With University certificate or diploma below bachelor level	-2.95	-7.08	-9.82	2.24	7.66	10.07	-4.30	-14.72	-18.38
With University certificate, diploma or degree at bachelor level or above	**-11.79**	**-26.50**	**-35.16**	**-13.74**	**-38.94**	**-47.33**	-3.83	-13.21	-16.54
65 years and over	**-8.72**	**-20.05**	**-27.02**	-5.15	-16.17	-20.49	-3.41	-11.81	-14.82
Average number of children in families	**12.12**	**32.41**	**48.47**	3.37	11.68	15.44	0.79	2.90	3.72
Living alone	**15.68**	**42.97**	**65.39**	**12.42**	**47.80**	**66.15**	7.59	30.41	40.31
Median amount of income	**-23.08**	**-47.47**	**-59.59**	**-19.53**	**-51.58**	**-61.03**	5.53	21.54	28.26
Non-official language as mother tongue	**11.35**	**30.17**	**44.94**	0.55	1.86	2.43	-3.33	-11.57	-14.52
Vaccination coverage (2nd dose)	**9.02**	**23.62**	**34.77**	-4.83	-15.24	-19.34	**-13.13**	**-39.98**	**-47.86**

The relative change of incidence rate are expressed as % given a one standard deviation change of the explanatory variables. The % of change due to vaccination coverage must be adjusted to account for the wave specific standard deviations to be comparable. Significant variables are in bold.
